# Previsional space during direct laryngoscopy: Implication in the difficult laryngoscopy: Erratum

**DOI:** 10.1097/MD.0000000000007996

**Published:** 2017-09-01

In the article, “Previsional space during direct laryngoscopy: Implication in the difficult laryngoscopy”,^[1]^ which appeared in Volume 96, Issue 27 of *Medicine*, an affiliation, “Seoul National University Medicine College, Jongno-gu, Seoul, Korea” was missing. Drs Sung-hee Han and Jin-hee Kim should have been affiliated with this institution in addition to the one already listed.
